# Development of an 8-valent *Salmonella* and *Shigella* MAPS vaccine

**DOI:** 10.3389/fimmu.2026.1886839

**Published:** 2026-07-27

**Authors:** Emily M. Boerth, Becky Roffler, Zoe Hancock, Joyce Gong, Calman A. MacLennan, Fan Zhang, Ying-Jie Lu

**Affiliations:** 1 Division of Infectious Diseases, Boston Children’s Hospital, Harvard Medical School, Boston, MA, United States; 2 Calman MacLennan Consulting Ltd, Abingdon-on-Thames, United Kingdom; 3 Department of Immunology and Immunotherapy, School of Medical and Dental Sciences, University of Birmingham, Birmingham, United Kingdom

**Keywords:** adjuvant, MAPS, *Salmonella*, *Shigella*, vaccine

## Abstract

**Introduction:**

*Salmonella* and *Shigella* infections are two of the leading causes of global diarrheal deaths in children younger than 5 years. Licensed vaccines against *Salmonella* Typhi have been shown to be effective, and other *Salmonella* and *Shigella* vaccine candidates are being evaluated in clinical trials.

**Methods:**

We have previously described two 4-valent vaccines against *Salmonella* and *Shigella* strains separately. Here, we tested the combination of *Salmonella* and *Shigella* vaccines in animal models. ELISA was used to determine antibody titers against antigens, and bactericidal and opsonophagocytic assays were used to determine the function of these antibodies.

**Results and discussion:**

We found that the 8-valent *Salmonella/Shigella* vaccine induced immune responses similar to those of the two 4-valent counterparts. The immunological benefits of the addition of the adjuvant aluminum phosphate (alum), R848/Alum, or AddaS03 were evaluated in rabbits. The results demonstrated that alum augments antibody production against vaccine protein components, and the addition of R848/Alum or AddaS03 significantly enhanced antibody production and bactericidal activity for some serotypes.

## Introduction

*Salmonella* and *Shigella* are bacteria that cause enteric and diarrheal diseases, which are primarily transmitted through the fecal-oral route, often via contaminated food, water, or person-to-person contact. These infections can also lead to invasive disease, with significant morbidity and mortality. As such, *Salmonella* and *Shigella* infections are major global public health concerns, particularly in lower- and middle-income countries (LMICs). The increasing prevalence of antibiotic-resistant strains presents a growing challenge for effective treatment worldwide. The Global Burden of Disease (GBD) 2021 report from the Institute of Health Metrics and Evaluation estimated that there were 9.3 million enteric fever caused by *S.* Typhi and *S.* Paratyphi A, which may have led to over 107,500 deaths (1). It also estimated 117,000 deaths from shigellosis, 81,800 of which were among children under five years of age (2). Infections with *Salmonella* and *Shigella* are also common among travelers and military personnel deployed in endemic areas (3).

Four key serovars of *Salmonella enterica* are responsible for a large majority of invasive *Salmonella* disease: *Salmonella enterica* serovars Typhi, Paratyphi A, Typhimurium, and Enteritidis. *S.* Typhi and *S*. Paratyphi A cause enteric (typhoid) fever and are highly endemic in parts of South Asia, Southeast Asia, and Sub-Saharan Africa. Invasive nontyphoidal *Salmonella* (iNTS) disease has a high associated case-fatality rate and particularly affects infants and immunocompromised individuals in sub-Saharan Africa. Globally, over 70% of all human isolates of iNTS are *S*. Typhimurium and *S*. Enteritidis (4). While a variety of *Shigella* serotypes can cause disease (5), *Shigella flexneri* 2a, 3a, and 6 and *Shigella sonnei* are the most common serotypes in LMICs (6). *Shigella sonnei* is mostly present in developed countries. A quadrivalent vaccine targeting *Shigella flexneri* 2a, 3a, 6, and *Shigella sonnei* is estimated to cover approximately 80% to 85% of global shigellosis cases (7).

Polysaccharide-protein conjugate vaccines against *Salmonella* Typhi (typhoid conjugate vaccines, TCV) are licensed and World Health Organization (WHO)-qualified, and there are currently several candidate vaccines targeting invasive non-typhoidal *Salmonella* (iNTS) and paratyphoid A in clinical trials. A bivalent Generalised Modules for Membrane Antigens (GMMA) outer membrane vesicle vaccine against *S*. Typhimurium and *S*. Enteritidis showed elevated anti-*S* Typhimurium and *S* Enteritidis O-antigen IgG and serum bactericidal responses against both bacteria in a phase I trial (8). A bivalent conjugate vaccine against *S*. Typhi and *S*. Paratyphi A was evaluated in a phase I study and elicited strong antibody responses to Vi and Paratyphi A O-specific polysaccharide (OSP) components (9). A trivalent vaccine containing the licensed TypbarTCV (Bharat Biotech, India), and *S.* Typhimurium, and *S.* Enteritidis OSPs conjugated to Flic generated greater than 4-fold increases in anti-Vi and anti-OSP antibodies in a phase I clinical trial (10). A detailed review of the current status of combination *Salmonella* vaccines was recently published (11).

There are currently no licensed vaccines against *Shigella*, but several, including live-attenuated, killed whole-cell and glycoconjugate vaccines, have been examined in clinical trials (reviewed in (12)). Historic Phase 3 efficacy trials of an *S. sonnei* conjugate vaccine in Israel demonstrated a strong association between IgG antibody against OSP and protective efficacy (13, 14). Serum IgG directed against the type III secretion system protein IpaB may also correlate with protection, as suggested by results from a study using a controlled human infection model of *Shigella* (15). Even though there are different age profiles of iNTS disease and typhoid fever, and a varied geographical distribution of *Salmonella* and *Shigella* disease, a single combination vaccine that could be used in all geographies is potentially attractive, as such a vaccine could be significantly cost-saving, offer greater opportunities for wide coverage of at-risk children, and require a shorter period of time to reach licensure and WHO prequalification.

Our group has previously described a novel vaccine technology, called MAPS (Multiple Antigen Presenting System), which allows for the incorporation of polysaccharides and proteins in affinity-linked complexes (16). The MAPS platform is particularly useful against serotype-diverse pathogens, as the antigens included can be modulated cost-effectively and with relative ease. MAPS vaccines have been shown to generate antibody responses both to polysaccharide and protein components in mice, rabbits, nonhuman primates and, more recently, in humans (16–26). The MAPS technology has been applied to a variety of pathogens, including *Streptococcus pneumoniae* (16, 23), *Staphylococcus aureus* (26), *Mycobacterium tuberculosis* (19), and *Salmonella* (20, 22). More recently, we have also evaluated a combination of IpaB and OSP in a quadrivalent MAPS *Shigella* vaccine. In animal models, this vaccine showed increased immunogenicity against the four included serotypes (18).

Here, we have combined our quadrivalent *Salmonella* MAPS vaccine (20) with the quadrivalent *Shigella* MAPS vaccine (18) to generate an 8-valent vaccine, aiming to evaluate antigen dose balance, effects of alum phosphate, and alternative adjuvant formulations in rabbits using binding and functional antibody assays. This MAPS vaccine incorporates pathogen-specific proteins and polysaccharides into a macromolecular complex using a biotin-rhizavidin interaction, as described previously (16). We find that the 8-valent vaccine elicits detectable immune responses to the antigens in rabbits, comparable to those of the two quadrivalent vaccines, and the immune responses were further enhanced by the use of adjuvants AddaS03 and R848/Alum.

## Results

### Optimization of the 8-valent MAPS polysaccharide concentration

We designed an 8-valent MAPS vaccine by generating eight monovalent MAPS vaccine components, each consisting of serotype-specific polysaccharides from *Shigella* and *Salmonella* (4 respectively) and two bacteria-specific fusion proteins. The *Shigella* components included IpaB as the fusion protein. The *Salmonella* components of the vaccine incorporated SseB. Both proteins are key virulence factors for each respective bacteria, and are highly conserved between serotypes. The four *Shigella* OSPs are from *S. flexneri* 2a, 3a, and 6, and *S. sonnei*. The four *Salmonella* polysaccharides are OSP from *S.* Typhimurium, *S.* Enteritidis, and *S*. Paratyphi A, as well as the capsular Vi polysaccharide of *S.* Typhi. Our previous multivalent MAPS work demonstrated that a two-dose immunization schedule generates optimal responses for both *Salmonella* and *Shigella* MAPS (18, 20). Among the eight serotypes we tested in each tetravalent presentation, we noted that some components induced smaller geometric mean fold-increases in antibody titers in rabbits, particularly *S* Enteritidis, *S*. flexneri 2a, and *S*. Paratyphi A OSPs. Conversely, *S. flexneri* 6 and *S. sonnei* OSPs generated strong immune responses after just one immunization and the second immunization did not increase the antibody titers. Thus, we first studied whether modifying the dose of these antigens in the 8-valent vaccine presentation would achieve more balanced immune responses between the different serotypes.

To this end, we tested four groups of doses as shown in **Table 1**: 5µg/polysaccharide (PS)/dose (group 1, the dose tested in our two quadrivalent vaccines), 1µg/PS/dose for *S. flexneri* 6 and *S. sonnei* and 5µg/PS/dose for remaining PS components (group 2), 10µg/PS/dose (group 3), and 10µg/PS/dose for *S. flexneri* 2a, *S.* Enteritidis, and *S.* Paratyphi A, and 5µg/PS/dose for the remaining PS components (group 4).

**Table 1:** Polysaccharide doses (µg/dose) by group.

Antigen Group	*Shigella*				*Salmonella*			
	*flexneri* 2a	*flexneri* 3a	*flexneri* 6	*sonnei*	Enteritis	Typhimurium	Typhi	Paratyphi A
**1**	5	5	5	5	5	5	5	5
**2**	5	5	1	1	5	5	5	5
**3**	10	10	10	10	10	10	10	10
**4**	10	5	5	5	10	5	5	10

**Figure 1:**
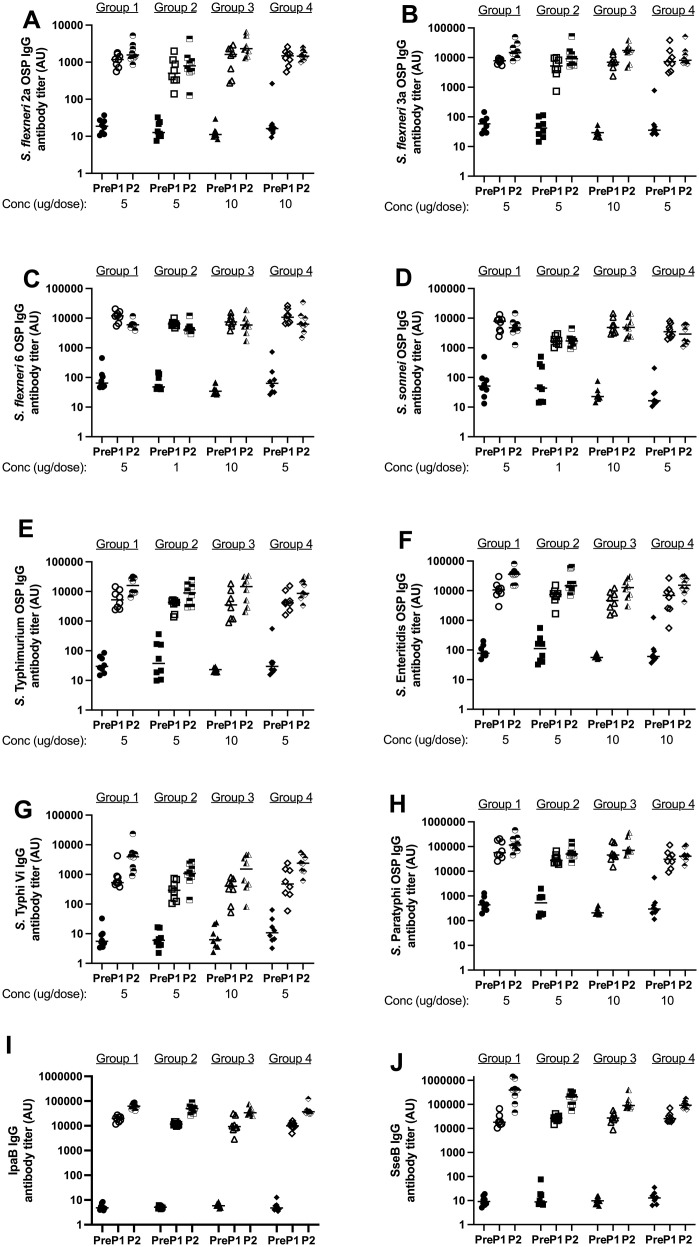
Evaluation of serum IgG antibody responses to polysaccharides (PSs and proteins following immunization of rabbits with two doses of different antigen concentrations of an 8-valent *Shigella*/*Salmonella* MAPS candidate vaccine. Pre, rabbit sera prior to MAPS immunization (closed symbols); P1, rabbit sera post one immunization (open symbols); P2, rabbit sera post two immunizations (half open symbols); Group 1, all rabbits received 5µg/PS/dose; Group 2, rabbits received 1µg/PS/dose of *S; flexneri* 6 and *S. sonnei* MAPS, and 5µg/PS/dose of the other 6 MAPS; Group 3; all rabbits received 10µg/PS/dose, Group 4; rabbits received 10µg/PS/dose of *S. flexneri* 2a, *S.* Enteritidis, and *S.* Typhi MAPS and 5µg/PS/dose of the other 5 MAPS. **(A)** *S. flexneri* 2a OSP IgG response. **(B)** *S. flexneri* 3a OSP IgG response. **(C)** *S. flexneri* 6 OSP IgG response. **(D)** *S. sonnei* OSP IgG response. **(E)** *S. Typhimurium* OSP IgG response. **(F)** *S. Enteritidis* OSP IgG response. **(G)** *S. Typhi* Vi IgG response. **(H)** *S. Paratyphi* A OSP IgG response. **(I)** IpaB IgG response. **(J)** SseB IgG response.

Rabbits were immunized twice and antibody responses were measured after the second immunization. As shown in **Figures 1A–H** and **Supplementary Table 2**, there was a detectable antibody response to all 8 PS antigens in each group. We also evaluated the antibody response to the two proteins (SseB and IpaB) and found that adjusting the dose of PS had less or no impact on antibody production against these protein components (**Figures 1I, J**). No clear advantage of higher or adjusted dosing was observed under the tested conditions. Thus, the 5µg/PS dose was used for all future experiments. We also compared the geometric mean fold rise for the 5µg/PS group to the previously published antibody results of the quadrivalent *Salmonella* and *Shigella* MAPS, and found that 8-valent MAPS had a similar antibody fold rise as the two previous vaccines.

### Role of alum in antibody responses

The role of alum in the vaccine formulation was evaluated by immunizing three groups of rabbits with the 8-valent vaccine under different conditions: without alum (no-adjuvant), with a standard concentration of alum (0.5 mg/mL; alum), and with a higher concentration of alum (1.25 mg/mL; alum high).

**Figure 2:**
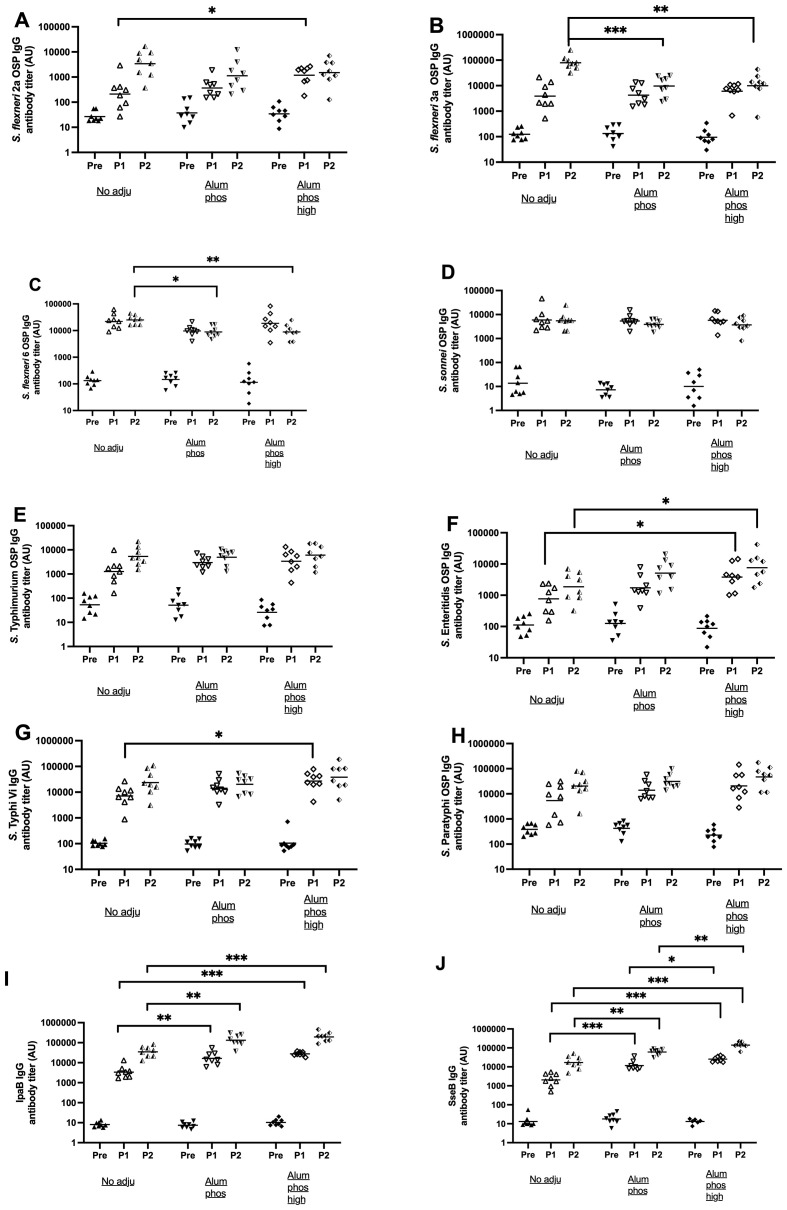
Evaluation of the effect of alum on the anti-PS and protein IgG responses following immunization of rabbits with two doses of an 8-valent *Shigella*/*Salmonella* MAPS vaccine. Pre: rabbit sera prior to MAPS immunization (closed symbols). P1, rabbit sera post one immunization (open symbols); P2, rabbit sera post two immunizations (half open symbols); Statistical analysis was calculated by Mann-Whitney U. Groups were compared to each other for the same blood draw timepoints. *P <0.05, **P<0.01, ***P<0.001. **(A)** *S. flexneri* 2a OSP IgG response. **(B)** *S. flexneri* 3a OSP IgG response. **(C)** *S. flexneri* 6 OSP IgG response. **(D)** *S. sonnei* OSP IgG response. **(E)** *S. Typhimurium* OSP IgG response. **(F)** *S. Enteritidis* OSP IgG response. **(G)** *S. Typhi* Vi IgG response. **(H)** *S. Paratyphi* A OSP IgG response. **(I)** IpaB IgG response. J. SseB IgG response.

The inclusion of alum had variable effects on antibody responses to *Shigella* OSPs (**Figures 2A–D**; **Supplementary Table 2**). After a single immunization, the alum high group produced a 5.6-fold (geometric mean) higher antibody response against *S. flexneri* 2a OSP compared to the no-adjuvant group (P < 0.05). However, after two immunizations, the highest antibody responses against *S. flexneri* 3a and *S. flexneri* 6 OSPs were observed in the no-adjuvant group. These responses were significantly higher than those in the alum and alum high groups (P < 0.001 and P < 0.01 for *S. flexneri* 3a; P < 0.05 and P < 0.01 for *S. flexneri* 6, respectively).

In contrast, alum enhanced antibody responses to *Salmonella* OSPs and Vi antigen (**Figures 2E–H**; **Supplementary Table 2**). After one immunization, the alum group showed approximately a 2-fold increase in antibody levels compared to the no-adjuvant group. The alum high group exhibited even greater increases, with antibody titers rising 2.7-, 5.0-, 3.8-, and 3.8-fold (geometric mean) against *S.* Typhimurium, *S.* Enteritidis, *S.* Typhi, and *S.* Paratyphi A, respectively. After two immunizations, antibody titers were generally similar across groups for three *Salmonella* MAPS antigens, except for *S. Enteritidis*, where titers remained elevated in the alum (2.7-fold) and alum high (4.1-fold) groups compared to the no-adjuvant group.

Antibody responses to IpaB and SseB were also enhanced by alum (**Figures 2I, J**). Following a single immunization, the alum group exhibited 4.8-fold (geometric mean) and 5.7-fold (geometric mean) increases in IpaB and SseB antibody levels, respectively, compared to the no-adjuvant group. This enhancement persisted after the second immunization, with 3.8-fold (IpaB, geometric mean) and 3.6-fold (SseB, geometric mean) increases. The alum high group showed even higher responses, with fold increases relative to the no-adjuvant group of 8.2 (post-dose 1) (geometric mean) and 5.5 (post-dose 2) (geometric mean) for IpaB, and 10.5 (post-dose 1) (geometric mean) and 8.2 (post-dose 2) (geometric mean) for SseB.

**Figure 3:**
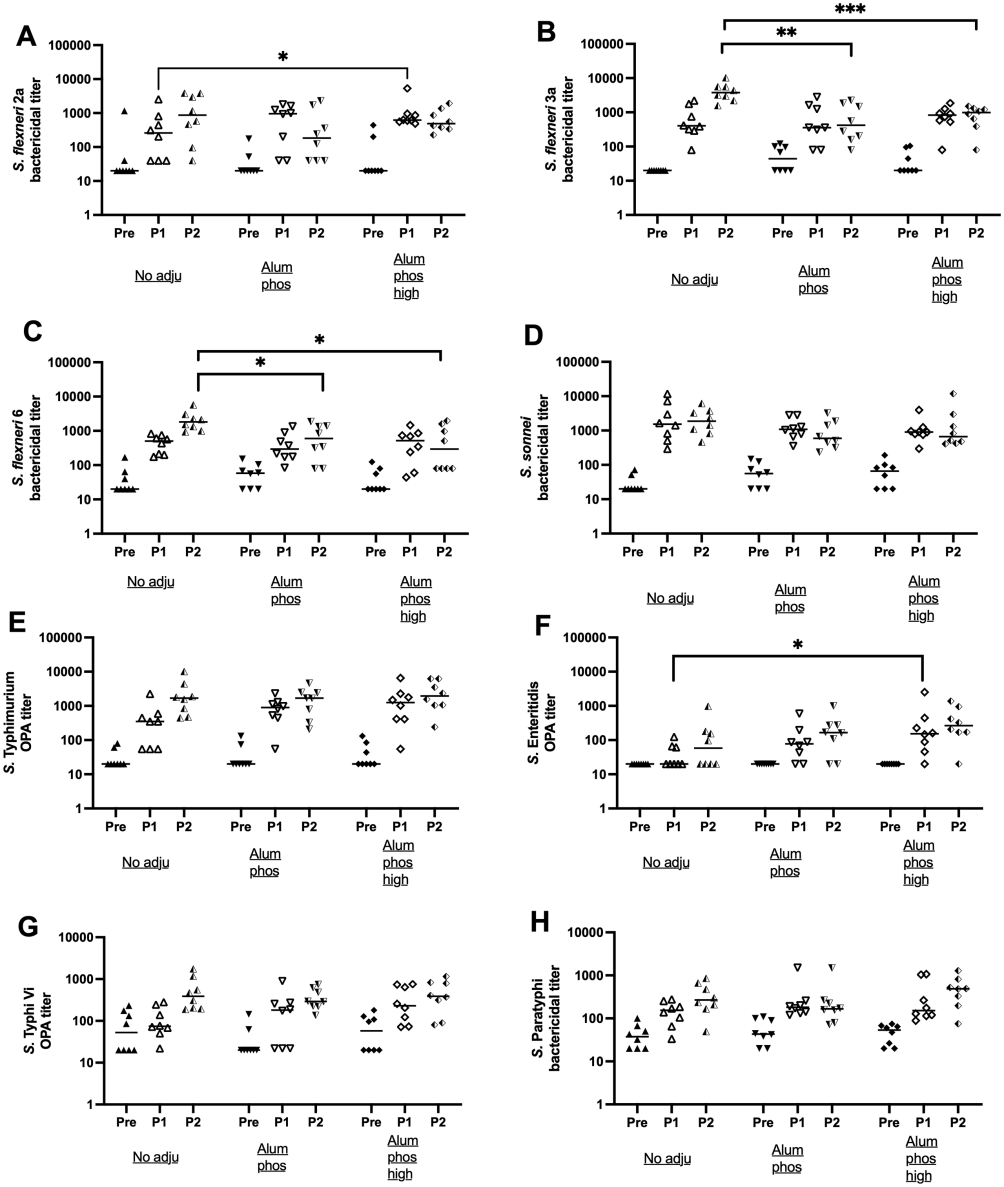
Evaluation of the effect of alum on the bactericidal activities following immunization of rabbits with two doses a candidate 8-valent *Shigella*/*Salmonella* MAPS vaccine. Pre: rabbit sera prior to MAPS immunization (closed symbols). P1, rabbit sera post one immunization (open symbols); P2, rabbit sera post two immunizations (half open symbols), Statistical analysis was calculated by Mann-Whitney U. Groups were compared to each other for the same blood draw timepoints. *P <0.05, **P<0.01, ***P<0.001. **(A)** *S. flexneri* 2a bactericidal titers. **(B)** *S. flexneri* 3a bactericidal titers. **(C)** *S. flexneri* 6 bactericidal titers. **(D)** *S. sonnei* bactericidal titers. **(E)** *S. Typhimurium* OPA titers. **(F)** *S. Enteritidis* OPA titers. **(G)** *S. Typhi* Vi OPA titers. (H) *S. Paratyphi* A bactericidal titers.

Serum bactericidal and OPA activity correlate with the antibody findings (**Figure 3**; **Supplementary Figure 2**). A mixed effect was observed for *Shigella*, consistent with the antibody response patterns. In contrast, bactericidal or OPA titers against *Salmonella* species were higher in rabbits immunized with alum-containing vaccines. After one immunization, the alum high group showed increases of 3.6-, 7.7-, and 3.1-fold (geometric mean) against *S.* Typhimurium, *S.* Enteritidis, and *S.* Typhi, respectively, compared to the no-adjuvant group. The alum group showed corresponding increases of 2.6-, 3.9-, and 2.4-fold (geometric mean). No significant change was observed for *S.* Paratyphi A.

### Effect of different adjuvants on antibody production against *S. flexneri 6* OSP

**Figure 4:**
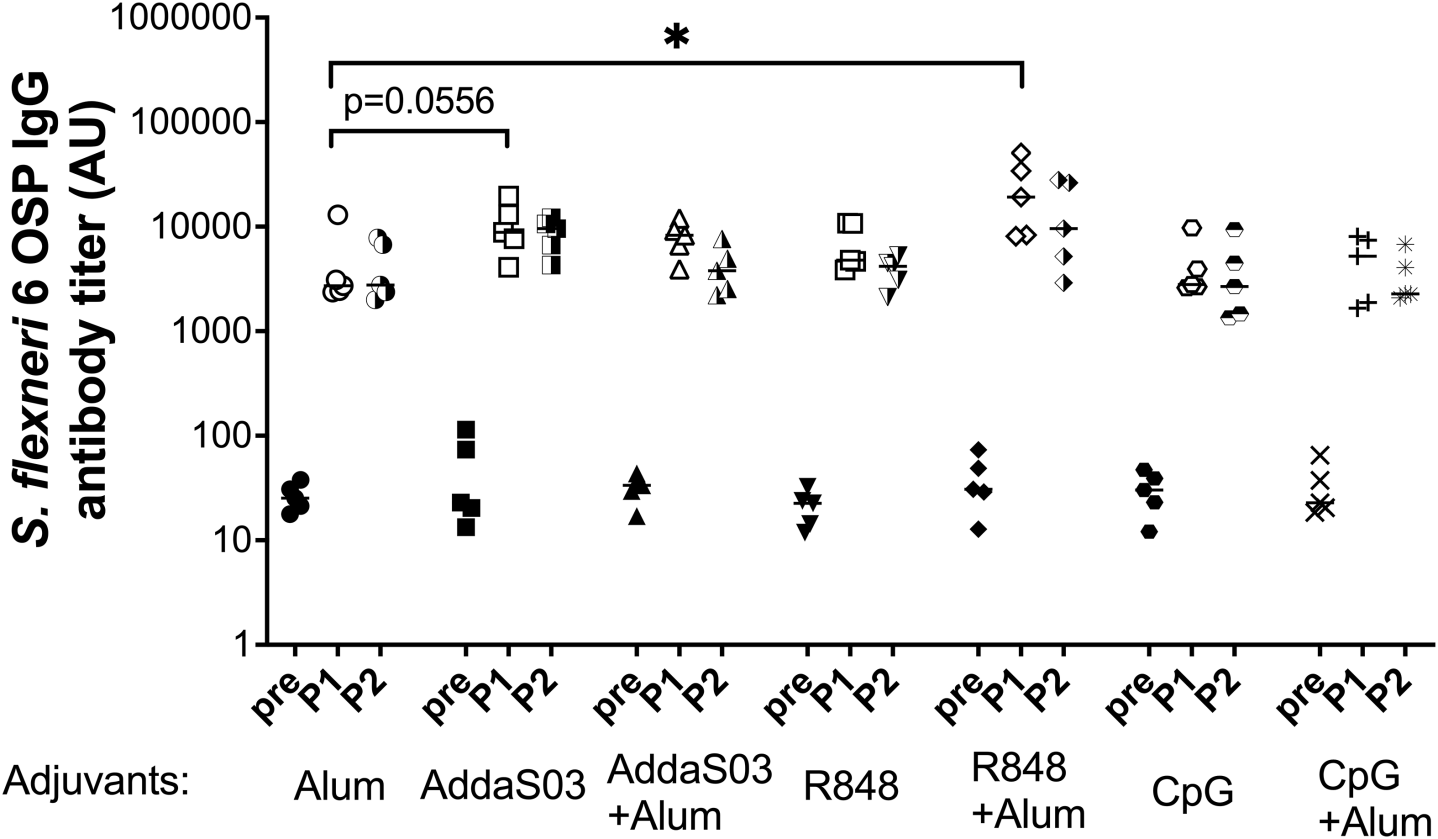
Effect of adjuvants on the serum IgG antibody directed against *S. flexneri* 6 OSP following immunization with two doses of monovalent *S. flexneri* 6 MAPS. *S. flexneri* 6 MAPS was formulated with different adjuvants and used for rabbit immunization. Pre: rabbit sera prior to MAPS immunization (closed symbols). P1; rabbit sera post one immunization (open symbols), P2; rabbit sera post two immunizations (half open symbols). Statistical analysis was calculated by Mann-Whitney U. Groups were compared to each other for the same blood draw timepoints. *P <0.05.

We noted that, with few exceptions (*S*. flexneri 3a, *S*. Typhimurium and *S.* Enteritidis), there was little evidence of enhancement of response between the first and second vaccine administration. To evaluate whether this represents a biological plateau compared with a lack of boosting, and to understand whether the primary antibody response could be increased, we evaluated the impact of different adjuvants on immunogenicity of the MAPS vaccine. Because changing the dose of *S. flexneri* 6 OSP did not affect the antibody production against OSP (**Figure 1C**), we evaluated the ability of three adjuvants to improve the immunogenicity of a monovalent *S flexneri* 6 MAPS vaccine: AddaS03, R848, and CpG in the presence or absence of alum. AddaS03 is an oil-in-water emulsion composed of squalene, polysorbate 80, and vitamin E, which has the same composition as AS03 (27, 28). R848 is a TLR7/8 agonist (29) that has been studied previously in preclinical applications for vaccines directed against influenza (30, 31), pertussis (32), and SARS-CoV-2 (33). CpG is a TLR 9 ligand (34), a version of which (CpG 1018) is a component of a recombinant Hepatitis B vaccine (35). We compared the IgG ELISA titer against *S. flexneri* 6 OSP (**Figure 4**; **Supplementary Table 2**) in sera from rabbits immunized with *S. flexneri* 6 MAPS formulated with these different adjuvants. While a detectable antibody response was noted in all groups, the groups including Addas03 and R848/Alum had the highest antibody titers, especially after the first immunization.

### Effect of Addas03 and R848/Alum on antibody production and function

**Figure 5:**
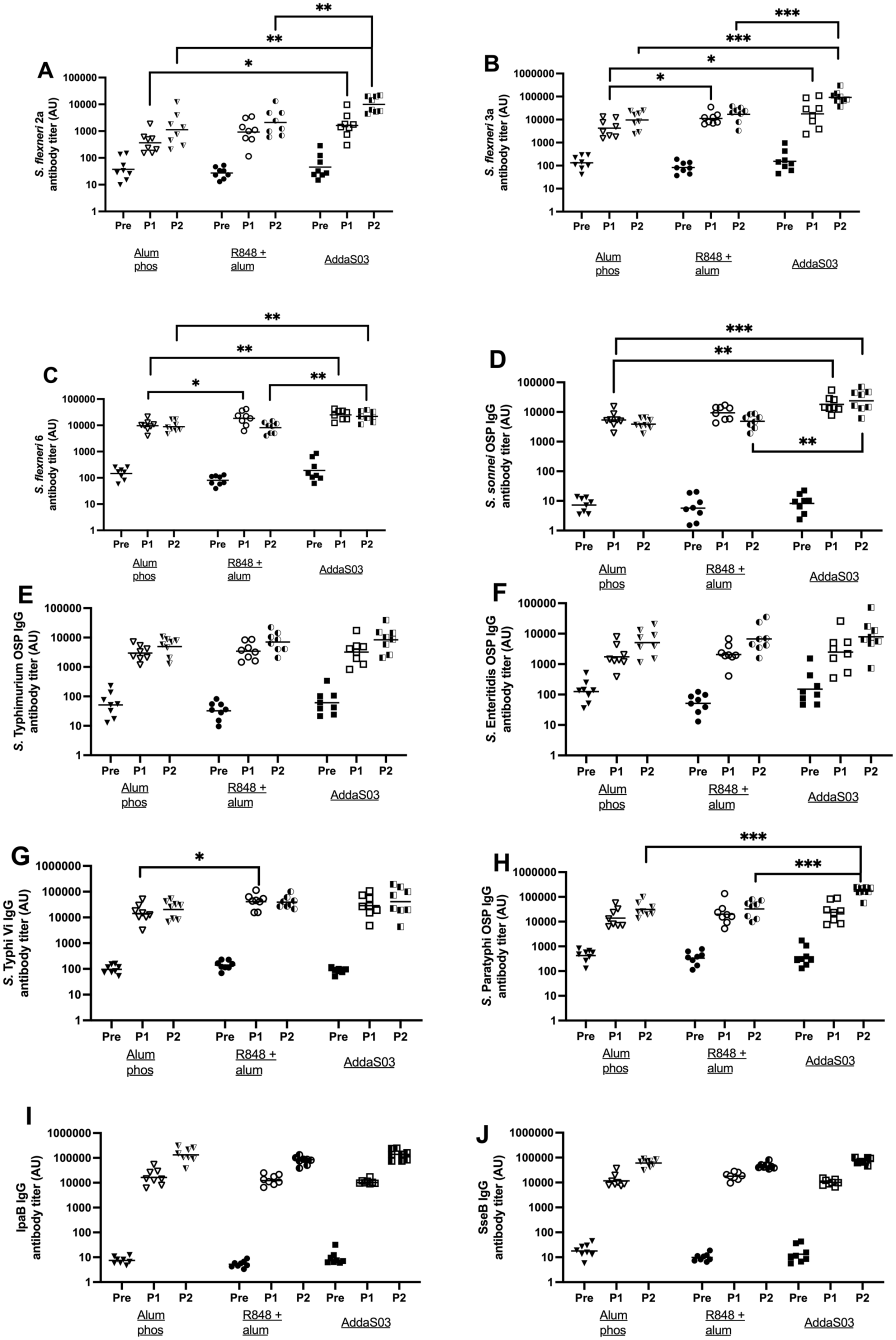
Evaluation of the effect of adjuvants on the serum IgG antibody production following immunization of rabbits with two doses of an 8-valent candidate *Shigella*/*Salmonela* MAPS vaccine. Pre: rabbit sera prior to MAPS immunization (closed symbols). P1: rabbit sera post one immunization (open symbols). P2: rabbit sera post two immunizations (half open symbols). Statistical analysis was calculated by Mann-Whitney U. Groups were compared to each other for the same blood draw timepoints. *P <0.05, **P<0.01, ***P<0.001. **(A)** S. flexneri 2a OSP IgG response. **(B)** S. flexneri 3a OSP IgG response. **(C)** *S. flexneri* 6 OSP IgG response. **(D)** *S. sonnei* OSP IgG response. **(E)** *S. Typhimurium* OSP IgG response. **(F)** *S. Enteritidis* OSP IgG response. **(G)** *S. Typhi* Vi IgG response. **(H)** *S. Paratyphi* A OSP IgG response. **(I)** IpaB IgG response. **(J)** SseB IgG response.

The results of *S. flexneri* 6 may not represent the behavior for other polysaccharides and proteins. We tested whether including Addas03 and R848/Alum in the 8-valent vaccine formulation would boost antibody production for the different components of the vaccine. Groups of 8 rabbits were immunized with 8V-vaccine formulated with R848/Alum or AddaS03, and compared with rabbits immunized with two doses of 8V-vaccine formulated with alum only. The concentration of serum IgG antibodies against each PS was assessed after each immunization (**Figure 5**; **Supplementary Table 2**). The combination of R848/Alum enhanced antibody production against *S. flexneri* 3, *S. flexneri* 6 OSP and Vi following one (P1) immunization (**Figures 5B, C, G**). AddaS03 enhanced antibody production for all *Shigella* OSPs, following both one (P1) and two (P2) immunizations (**Figures 5A–D**). The post 2^nd^ immunization (P2) antibody titers for *Shigella* OSPs in the AddaS03 group were higher than those of the R848/Alum group. The antibody responses against *Salmonella* OSPs and Vi were not enhanced by the addition of AddaS03, except for the P2 responses of *S.* Paratyphi A OSP (**Figure 5H**). Using R848/Alum and AddaS03 as adjuvants had no statistically significant enhancement on serum antibody production against IpaB and SseB (**Figures 5I, J**).

**Figure 6:**
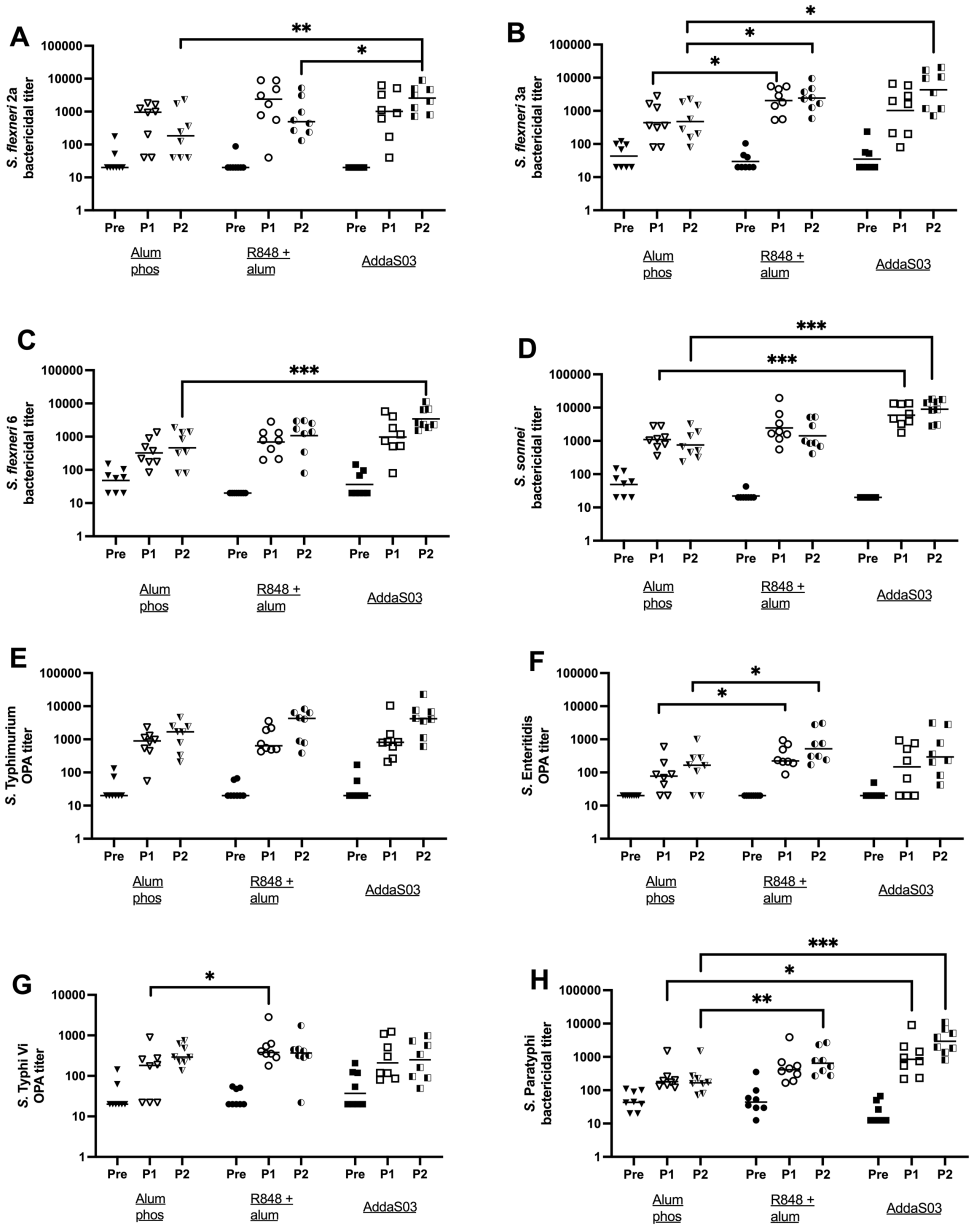
Evaluation of the effect of adjuvants on the serum bactericidal titer response following immunization with an 8-valent *Shigella*/*Salmonella* MAPS candidate vaccine. Pre; rabbit sera prior to MAPS immunization (closed symbols), P1; rabbit sera post one immunization (open symbols), P2; rabbit sera post two immunizations (half open symbols). Statistical analysis was calculated by Mann-Whitney U. Groups were compared to each other for the same blood draw timepoints. *P <0.05, **P<0.01, ***P<0.001. **(A)** *S. flexneri* 2a bactericidal titers. **(B)** *S. flexneri* 3a bactericidal titers. **(C)** *S. flexneri* 6 bactericidal titers. **(D)** *S. sonnei* bactericidal titers. **(E)** *S. Typhimurium* OPA titers. **(F)** S. Enteritidis OPA titers. **(G)** *S. Typhi* Vi OPA titers. **(H)** *S. Paratyphi* A bactericidal titers.

We then analyzed the sera for bactericidal and OPA activity. A similar trend of enhancement by the addition of the new adjuvants was observed. R848/Alum increased bactericidal titers for *S*. flexneri 3 (**Figure 6B**) and *S*. Paratyphi A (**Figure 6H**), OPA titers for *S*. Enteritidis (**Figure 6F**) and *S*. Typhi (**Figure 6G**). Addas03 increased bactericidal titers for all *Shigella* strains (**Figures 6A–D**) and *S*. Paratyphi (**Figure 6H**). ELISA titers generally correlate well with either BCA or OPA titers but vary among serotypes (**Supplementary Figure 1**).

## Discussion

We have previously shown that a quadrivalent *Salmonella* MAPS and quadrivalent *Shigella* MAPS candidate vaccine elicit strong serum IgG responses to each serotype after immunization of rabbits (18, 20). Building on these findings, we have formulated and evaluated an 8-valent *Shigella*/*Salmonella* MAPS candidate vaccine containing the combined components from both quadrivalent vaccines. We demonstrate that this combination vaccine elicits increased serum IgG antibody responses in rabbits against each of the antigen components. We also show that including alum has a mixed effect: it enhanced antibodies to certain *Salmonella* polysaccharides and proteins, but reduced antibodies to some *Shigella* OSPs after two immunizations. The immune responses can be further enhanced by formulation with two novel adjuvants (R848 and Addas03) in the presence or absence of alum.

We have observed that several components of our vaccine had no boosting effect after the second immunization, even when we changed the dose or added adjuvants other than alum. This could be due to several factors, such as antigen dose excess, limitations of the rabbit model, or perhaps an immunization schedule that is too shortened to observe a stronger effect of a booster dose. As rabbit immunogenicity studies may not reflect human responses, a careful design of dose and immunization schedule should be considered in human clinical trials.

Combination vaccines have significant advantages over monovalent vaccines, as they can significantly reduce vaccine costs and simplify the vaccine schedule (36). For LMICs, combination vaccines reduce the cost of needles and syringes, cold chain storage, inventory management, and vaccination visit capacity, making combination vaccines cheaper and quicker to administer. In the case of *Salmonella* disease, the licensing of the first TCV, TypbarTCV (37), has led to the evaluation of dual combinations of Vi and paratyphi A conjugate vaccines, which were recently tested in human trials (9–11). Other considerations include combining the *Salmonella* multivalent vaccines with a licensed commercial vaccine, such as the pneumococcal or meningococcal conjugate vaccines.

This study tested the possibility of combining the *Salmonella* and *Shigella* vaccines, by evaluating performance in animal models. The potential future benefits of combining *Salmonella* and *Shigella* vaccines include the geographic overlap between the diseases as well as their target populations. The Global Burden of Disease 2021 Enteric and Diarrheal Disease Burden Estimates in Children <5 Years listed *Salmonella* and *Shigella* infections as the leading disease-causing agents, alongside rotavirus infection. Thus, a vaccine targeting both bacteria may have a dramatic impact on morbidity and mortality in children in LMICs.

However, there are also other factors to consider, such as age of administration, dose scheduling, regulatory hurdles and cost of production for a combination vaccine. *Salmonella* infections are highly prevalent in children under 5 years old, particularly iNTS disease, predominantly caused by *S.* Typhimurium and *S*. Enteritidis, which has a peak incidence around 1 year of age (38). Similarly, Shigellosis has the highest incidence among children aged less than 5 years old, peaking at 12 to 24 months of age (39). Thus, immunization at an early age (e.g. 6 months) with a booster dose at 12 months of age might be necessary for the 8-valent vaccine in human trials (11).

This schedule, or some variant thereof, might also help the durability of the immune responses to the Vi polysaccharide. Reports have shown that a single injection of the licensed TCV, TypbarTCV, in three phase 3 randomized clinical trials conducted in Malawi, Nepal, and Bangladesh, had good short-term protective efficacies, but two of the three trials showed a decline in protection from over 80% to ~55% in long-term follow-up studies (40–42). The reason for the decline in protection remains unknown, but suggests that changes are needed to the vaccination schedule to improve the long-term protective efficacy of TCVs. A recent trial showed that a 2-dose regimen of TypbarTCV achieved a higher seroconversion rate when the second dose was given at 15 months rather than at 12 months (43). A study in Malawi showed that a booster dose at a 4-year interval elicited an antibody response higher than that in children receiving TypbarTCV for the first time (44). The responses to the booster dose were higher at 160–180 days after immunization than in those who received the first dose. Thus, including TCV in the *Shigella*/*Salmonella* 8-valent candidate vaccine, administered using a 2-dose regimen might be beneficial for extending the duration of protection afforded by TCV.

Novel adjuvants are often used to enhance immune responses to vaccine candidates (45). AS03 has been used in a few vaccines targeting viral antigens (46–48), and a recent study showed that including AS03 in a tetravalent *Klebsiella pneumoniae* OSP conjugate vaccine enhanced IgG production against 3 of 4 OSPs in a human trial (49). We tested various adjuvants for their ability to enhance antibody responses to OSP and Vi in this study. Using AddaS03 enhanced IgG antibodies to all *Shigella* OSP and one *Salmonella* OSP (**Figure 5**). Even though the proposed mechanism of action of AddaS03 is similar to that of AS03: enhancing the presentation of antigen signals on the surface of antigen-presenting cells (APCs) through the slow release of antigens and stimulation of APCs with squalene (28), it should be pointed out that AddaS03 is not a clinical grade material and for research purposes only. It was used here to evaluate whether this type of adjuvant could enhance the immune responses to the vaccine. We confirmed here that alum is required for optimal production of IgG antibody to the protein antigens in our 8-valent vaccine, and that OSP IgG responses to some OSPs can be further enhanced with the addition of R848. However, we have also observed that changing adjuvants may also reduce immune responses to some antigens, which suggests that the interaction of antigen with different adjuvants might require a carefully designed study for each antigen with the adjuvant, or there might be some interference between antigens when different adjuvants are used.

A few limitations in this study should be mentioned. One limitation is that we have only studied the vaccine in the rabbit model, which may not accurately reflect infant responses. We did not evaluate the duration of the immune responses and the assessment of the mucosal immune responses, which are important for the protection against *Salmonella* and *Shigella*. The study only performed limited physical/chemical analysis of different formulations and further characterization of these formulations is needed. A formal stability and toxicology study is also required to move the candidate vaccine beyond preclinical stages. Finally, adjuvants other than alum could raise concerns such as safety, reactogenicity, regulatory acceptability in infants, and compatibility with pediatric schedules. The availability of these adjuvants for LMIC childhood immunization programs should also be carefully considered.

In conclusion, our 8-valent *Salmonella*/*Shigella* candidate MAPS vaccine induced increased immune responses, that could be augmented by the addition of alum, AddaS03 and R848 adjuvants. Such a combination vaccine, if immunogenic in infants and children, may represent an attractive option for controlling *Salmonella* and *Shigella* disease in LMICs. Further studies are needed to characterize formulation, stability, toxicology, durability, and mucosal immunity of the vaccine before eventually testing the vaccine in controlled human studies and infants.

## Materials and methods

### Bacterial strains and reagents

*Shigella flexneri* strains were obtained from Dr. Sharon Tennant from the University of Maryland. *Shigella sonnei* 53G was obtained from Dr. Eileen Barry from the University of Maryland. NTS strains were obtained from the Global Enteric Multicenter Study collection from Dr. Sharon Tennant at the University of Maryland. Aluminum phosphate was obtained from Croda (Plainsboro, NJ, USA). 1-cyano-4-dimethylaminopyridiniumtetrafluoroborate (CDAP) was purchased from Thermo Fisher (Waltham, MA, USA). T7 Shuffle expressing competent cells were purchased from New England Biolabs (Ipswich, MA, USA). AddaS03, R848 and CpG were purchased from InvivoGen (San Diego, CA, USA). Baby rabbit complement was obtained from Pel-Freez Biologicals (Rogers, AR, USA). All other reagents were obtained from Sigma (St. Louis, MO, USA).

### Vaccine preparation and immunization procedures

The *Salmonella* and *Shigella* MAPS vaccines were manufactured as described previously (18, 20). Briefly, OSP and Vi were biotinylated with CDAP to have a 0.01-0.03 mg/mg biotin/polysaccharide ratio. Rhavi-IpaB and Rhavi-SseB were added to each of the biotinylated polysaccharide separately with a 5:1 protein:polysachharide ratio. Excess free proteins were separated from MAPS using size-exclusion chromatography. The final MAPS vaccines have a protein:polysaccharide ratio between 3:1 to 5:1. Protein concentration was determined by the BCA method (Pierce), and Vi concentration was determined by the acridine orange method (50). OSP concentration was determined using the Anthrone method (51). The monovalent MAPS were combined to form multivalent MAPS as indicated in each experiment.

MAPS vaccines were combined with alum at 0.5mg/mL in a 5mL tube for all experiments except the adjuvant formulation studies. Vaccines in these studies had the following adjuvant ratios. R848/Alum was 20ug R848/dose and 0.5mg/ml alum. AddaS03 was 1:1 (v:v) of adjuvant to vaccine. All vaccines were formulated in 20 mM Histidine buffer, pH 6 with 150 mM NaCl, and 0.02% polysorbate 80. Regular alum was used at 0.5 mg/ml. Alum high dose (alum high) was at 1.25mg/ml. All vaccines were formulated to have 5 μg of each polysaccharide in a 0.5 ml injection, except for the dose studies in **Figure 1**. Vaccines were rotated overnight at 4 °C. Rabbit immunization experiments were performed at Cocalico Biologicals Inc. (Denvers, PA, USA). New Zealand white rabbits (average weight 5–6 lbs, male) were randomized by the staff at the animal facility. Rabbits were immunized on days 0, 21 intramuscularly with MAPS vaccine in a 0.5 ml injection. Blood draws (5 ml serum) were collected before each immunization and at day 42. Euthanasia of rabbits is completed using intravenous injection of Euthaphen euthanasia solution in a dosage of 1ml for each 10 lbs. (4.5kg) of body weight, per the manufacturers and veterinary recommendations. This is an overdose of pentobarbital sodium in combination with phenytoin sodium and is consistent with the recommendations of the Panel on Euthanasia of the American Veterinary Medical Association. Procedures in the studies were in compliance with the National Institutes of Health guidelines and were approved by the Institutional Animal Care and Use Committee. Cocalico Biologicals, Inc is registered with the United States Department of Agriculture (23-B-0028 and 23-R-0089) and has an approved animal welfare assurance with the National Institutes of Health Office of Laboratory Animal Welfare (D16-00398 (A3669-01)).

### Adsorption rate of vaccines

Unbound MAPS vaccines were separate from alum for vaccines containing alum formulation by spinning at 10,000 *g* for 5 minutes and carefully transferring the supernatant fraction to a new tube. The protein concentrations from supernatants and the original vaccines were determined by BCA method. The percentage of adsorption was calculated by (original concentration-supernatant concentration)/original concentration×100. The adsorption rate for the alum formulation was at 71.5%; the adsorption rate for the alum high formulation was at 77%; the adsorption rate for the R848/Alum formulation was at 73%.

### Enzyme-linked immunosorbent assay

IgG antibody titers against all OSPs, Vi PS, and both fusion proteins were measured using methods previously described (52, 53). All procedures were performed at room temperature. Briefly, NUNC Maxisorp plates were coated with 100µL of 1µg/mL of protein or 1µg/mL of biotin-polysaccharide/Egg avidin in PBS overnight. There is no cross-reactivity between Egg avidin and Rhizavidin, so it will not introduce background signals from antibodies against Rhizavidin. Plates were washed with PBS Tween (PBST) and blocked with 200µL of PBS 1% BSA for 1 hour. Rabbit serum was serially diluted 1:3 starting at 1:50 in 100µL PBST and plates were incubated for 2 hours. Plates were washed with PBST and incubated with anti-rabbit IgG Fc HRP conjugate antibody (Jackson ImmunoResearch Catalog #111-035-046) diluted 1:10,000 in PBST for 1 hour. Plates were washed a final time then developed with Sureblue. Reaction was terminated with 1M HCl and absorbances were read at 450nM. A standard serum obtained from previous immunizations using the monovalent vaccine was used for each antigen-coating throughout the whole experiment. Arbitrary units were defined for each antigen by assigning the standard serum to a titer of 10,000.

### Serum bactericidal assay

Activity of the antisera against *Shigella* strains and *S.* Paratyphi was studied with SBAs described previously with some modifications (54). Briefly, dilutions of heat-inactivated rabbit sera were incubated with the relevant bacteria and baby rabbit complement for two hours at 37 °C. Each well was plated in triplicate on blood agar plates and colonies were counted to determine number of bacteria that survived the killing activity. The inverse of the lowest concentration of serum where 50% of the killing occurred was defined as the killing titer.

### Opsonophagocytic assay

Activity of the antisera against *S.* Typhimurium, *S.* Enteritidis, and *S.* Typhi was analyzed using OPAs described previously with some modifications (20, 22). Briefly, dilutions of heat inactivated rabbit sera were incubated with the relevant bacteria to allow antibodies to bind. Next, baby rabbit complement and differentiated HL60 cells were added to each well. The reaction was terminated, and samples were plated in triplicate on blood agar plates. Colonies represented the bacteria that survived the killing activity. The OPA titer was determined in the same manner as the SBAs.

### Statistical analysis

Statistical analyses were carried out using the unpaired nonparametric Mann-Whitney U test in PRISM (version 10.0, GraphPad Software, Inc). Each group was compared to the control group at the same time point of blood draw. Non-detectable titers were assigned the lowest detection limit, which is half of the antibody concentration of the lowest standard used in the plate multiplied by the dilution factor of the sample.

## Data Availability

The original contributions presented in the study are included in the article/**Supplementary Material**. Further inquiries can be directed to the corresponding author.

## Supplementary material

Supplementary Figure 1 Correlation of IgG antibody titers to bacterial killing activity. Data were from P1 and P2 sera from **Figures 2**, **3**, **5** and **6**.
