# Supplementary material for: Development of an 8-valent Salmonella and Shigella MAPS vaccine
Source: Front Immunol. 2026 Jul 27;17:1886839. doi: 10.3389/fimmu.2026.1886839 (PMC13454105; doi:10.3389/fimmu.2026.1886839)
Supplement: Supplementary file 1 [file Table1.docx]

| Vaccine  Antigen | 4V Shigella ^1^ | 4V Salmonella ^2^ | 8V vaccine ^3^ |
| --- | --- | --- | --- |
| *S. flexneri 2a OSP* | 41.3 |  | 97.6 |
| *S. flexneri 3a OSP* | 87.0 |  | 305.4 |
| *S. flexneri 6 OSP* | 53.2 |  | 71.0 |
| *S. sonnei OSP* | 120.7 |  | 82.6 |
| *S. Typhimurium OSP* |  | 679.3 | 448.6 |
| *S. Enteritidis OSP* |  | 97.0 | 368.6 |
| *S. Typhi Vi* |  | 75.5 | 493.9 |
| *S. Paratyphi OSP* |  | 168.1 | 276.0 |
| IpaB | 6000.3 |  | 11752.2 |
| SseB |  | 4252.9 | 34488.5 |

Supplemental Table 1 Geometric mean fold rise of antibody titers after two immunizations from different MAPS vaccines

1. The Geometric mean fold rise (GMFR) is calculated by dividing the geometric mean of post 2 immunization titers by the geometric mean of pre-immunization titers. GMFR of 4V Shigella MAPS data was from Figure 5 of Boerth et al. [1]; 2. The GMFR of 4V Salmonella MAPS data was calculated from Figure 4 of Boerth et al. [2]; 3. The GMFR of 8V Salmonella/Shigella Vaccine was calculated from group 1 (5 μg dose, same as the previous studies) of Figure 1 from the current study.

1.	Boerth EM, Gong J, Roffler B, Hancock Z, Berger L, Song B, et al. Evaluation of a Quadrivalent Shigella flexneri Serotype 2a, 3a, 6, and Shigella sonnei O-Specific Polysaccharide and IpaB MAPS Vaccine. Vaccines (Basel). 2024;12(10). Epub 20240924. doi: 10.3390/vaccines12101091. PubMed PMID: 39460258; PubMed Central PMCID: PMCPMC11510904.

2.	Boerth EM, Gong J, Roffler B, Thompson CM, Song B, Malley SF, et al. Induction of Broad Immunity against Invasive Salmonella Disease by a Quadrivalent Combination Salmonella MAPS Vaccine Targeting Salmonella Enterica Serovars Typhimurium, Enteritidis, Typhi, and Paratyphi A. Vaccines (Basel). 2023;11(11). Epub 20231031. doi: 10.3390/vaccines11111671. PubMed PMID: 38006003; PubMed Central PMCID: PMCPMC10675568.
